# Proton pump inhibitor prescription patterns in hospitalized patients before and after targeted intervention

**DOI:** 10.3389/fphar.2026.1838223

**Published:** 2026-07-07

**Authors:** Johny Gerjy Salem, Sarah Jalloul, Ahmad Hammoud, Jihane Atrash, Karim Halabi, Melissa Alkatrib, Mira Sabat, Louis Chaptini

**Affiliations:** 1 Department of Gastroenterology, Faculty of Medicine, University of Balamand, El Koura, Lebanon; 2 Department of Internal Medicine, Faculty of Medicine, University of Balamand, El Koura, Lebanon; 3 Department of Mathematics, Faculty of Arts and Sciences, University of Balamand, El Koura, Lebanon; 4 Department of Medicine, Digestive Diseases, Yale School of Medicine, New Haven, CT, United States

**Keywords:** inappropriate prescription, Lebanon, overuse, proton pump inhibitor, stewardship

## Abstract

**Background:**

The increasing utilization of proton pump inhibitors (PPIs) in patients without clear medical indications has raised concerns regarding potential risks, highlighting the importance of de-prescription. Ensuring the appropriate use of PPIs is an important hospital patient safety measure. The aim of this study is to describe the impact of a targeted quality improvement intervention on the use of PPIs in patients admitted to the hospital.

**Methods:**

This cross-sectional study consisted of two phases looking at PPI prescription in patients admitted to a community hospital in Lebanon. A total of 400 patients were included, with 200 patients in each phase. An educational non-coercive intervention was implemented between the phases to promote the understanding of the appropriate indications for PPI prescription. The primary outcome was the change in PPI overuse between phase I and phase II, defined as PPI prescribing among patients that are admitted without a valid indication for its use. To control for confounding, a multivariable logistic regression was performed adjusting for age, sex, and hospital ward.

**Results:**

During phase I, 84% of patients were prescribed a PPI upon admission, compared with 78.5% during phase II. Among patients with no valid indication for PPI prescription, 77.5% received PPI in phase I versus 69.3% in phase II (reflecting PPI overuse). This difference was not statistically significant (p = 0.18). After adjusting for age, sex, and ward category, the difference in inappropriate PPI prescription between the two phases remained statistically non-significant (adjusted OR = 0.911, 95% CI: 0.454–1.82, p = 0.792). Among patients with valid indication for PPI prescription, 10.8% did not receive PPI in phase I versus 5.5% in phase II (reflecting underuse). This difference was not statistically significant (p = 0.21).

**Conclusion:**

This study demonstrated that a non-coercive targeted educational intervention did not significantly reduce PPI overuse among hospitalized patients, even after controlling for the confounding variables. More rigorous and sustained educational efforts, together with improved adherence to prescribing guidelines, may be necessary to achieve statistically significant results.

## Background

Proton pump inhibitors (PPIs) are one of the most widely prescribed drugs worldwide due to their efficacy, safety, and tolerance. This class of medication is used for the treatment of acid-related disorders. The increasing prescription of PPIs in patients without clear medical indications has raised concerns regarding the potential risks, highlighting the importance of de-prescription. Many studies have shown that PPIs are frequently used inappropriately both in inpatient and outpatient settings (Souaid et al., 2020; Nasser et al., 2010; Hoteit et al., 2020; Soubra and Issa, 2019; Sheikh-Taha et al., 2012; Al-Dosari et al., 2021; Lenoir et al., 2019; Shah et al., 2023; Naunton et al., 2000; Gamelas et al., 2019; Shin, 2015; Alosaily et al., 2023; Alam et al., 2025; Elli et al., 2025; Steinsdóttir et al., 2024). Ensuring the appropriate use of PPIs is an important hospital patient safety and quality improvement initiative. Numerous interventions have been described (Wiggins et al., 2023; Baiardi et al., 2023; Barraquer and Roy, 2023; Del Giorno et al., 2018; Skalli et al., 2015; Targownik et al., 2022; Palmowski et al., 2024), leading to different degrees of success in decreasing the inappropriate prescription of these medications. This study aims to describe the impact of a targeted quality improvement intervention on the inappropriate use of PPI in patients admitted to the hospital.

## Materials and methods

This study was conducted in compliance with the ethical standards of the responsible institution on human subjects and with the Helsinki Declaration. Institutional Review Board (IRB) was obtained from the IRB committee of the hospital. This cross-sectional study consisted of two phases looking at PPI prescription in patients admitted to different wards of the hospital. The first phase was from March to June 2024, and the second phase was from July to September 2024. The primary outcome was the change in PPI overuse between phase I and phase II, which is defined as PPI prescribing among admitted patients without a valid indication for its use. PPI underuse, defined as the failure to prescribe PPI in patients with a valid indication, was also assessed as a secondary outcome.

### Intervention

Two weeks before the second phase, a targeted educational intervention was implemented for medical staff involved in prescribing PPIs in the hospital. Emails were sent to the admitting physicians, hospitalists, residents, nurses, and hospital pharmacists to introduce the research project and invite them to attend an educational session. The session was delivered as an interactive presentation of approximately 30 min, facilitated by two members of the gastroenterology department (an attending physician and a gastroenterology fellow). A total of 20 staff members attended the session, including hospitalist physicians, internal medicine, and surgery residents at different levels of training, along with nurse managers and clinical pharmacists. The presentation provided an overview of the appropriate indications for PPI use in the hospital setting, including a history of peptic ulcer disease, severe GERD or erosive esophagitis, Barrett’s esophagus, eosinophilic esophagitis, peptic stricture, NSAID use with gastrointestinal bleeding risk factors, dual antiplatelet therapy with gastrointestinal bleeding risk factors, ICU stress ulcer prophylaxis in patients with major risk factors, and Zollinger–Ellison syndrome. Several scenarios involving PPI misuse, including overuse and underuse, were also discussed to reinforce understanding. In addition, prior to implementation, one-on-one sessions were conducted by the same investigators on the wards targeting staff who were not able to attend the presentation (nurses and residents). The educational content was structured around a de-prescribing algorithm adapted from the American Gastroenterological Association (AGA) Clinical Practice Update on de-prescribing of PPIs by Targownik et al. (2022) covering evidence-based indications for initiating, continuing, and discontinuing PPI therapy in the hospital setting. Each participant received a written summary of the algorithm for reference. Assessment of participants’ knowledge or comprehension of the educational material was not included as part of the intervention; therefore, no post-intervention surveys, quizzes, or formal assessments were administered. The study was designed to evaluate the impact of a single educational exposure on subsequent PPI prescribing behavior.

### Inclusion criteria and data collection

A total of 400 patients were included, 200 patients in each phase. The population included consecutive patients 18 years and above, admitted to medicine, intensive care unit (ICU), cardiac care unit (CCU) and surgery wards. Data were collected from the electronic medical file of each patient by independent investigators. Collected information included age, sex, length of stay, reason for admission, past medical history, past surgical history, home medications, indication for PPI at admission, PPI type, dose, and duration of treatment. Data were collected anonymously using Excel and subsequently transferred to a dedicated statistical software program for analysis (R version 4.2.2 and RStudio). Patients were excluded if their data were incomplete or if their hospital admission was interrupted because of transfer to another institution or death.

### Statistical analysis and sample size calculation

Taking into consideration the primary outcome, a pilot study was conducted on 50 admitted patients. Out of those patients, 43 (86%) had no valid indication to be prescribed PPI, yet 59.5% out of those were prescribed the medication. The sample size required to reach the first proportion (86%) within a margin of error of 0.05 and a significance of 0.05 in phase I is 185. Within a level of significance of 0.05 and a power of 80%, the sample size required to detect a significant difference of 15% in the proportion of prescribed but not indicated patients is 170 for each of the two phases. This number represents, as per the pilot study, 86% of the total sample size. As a result, the total sample size was set at 200 patients per phase.

The data were analyzed using R version 4.2.2 and RStudio. The main variable of interest was the proportion of PPIs prescribed without a valid indication before and after the intervention. For each binomial distribution, a point estimate was calculated, the normal approximation was verified, and the 95% confidence interval and p-value were computed for the difference between the proportions.

The primary analysis included patients without a valid PPI indication at admission (n = 216). The primary outcome was the rate of PPI prescription among the admitted patients without a valid indication for its use (reflection of overuse). The main independent variable was the study phase (phase I = reference). Additional analysis included the rate of failure to prescribe PPI in patients with a valid indication. The model was adjusted for age, sex, and ward category. Because some individual ward categories had small or empty subgroups, a simplified ward classification grouping patients into surgery versus non-surgery wards was used for the final adjusted model. The adjusted odds ratios (aORs), 95% confidence intervals (CIs), and p-values were reported. Statistical significance was set at p < 0.05.

## Results

A total of 200 patients were included in each phase. The mean age in phases I and II was 62.38 and 54.26 years, respectively. Gender distribution was balanced in both the phases. The distribution of patients across different hospital wards was markedly different between the two phases: the surgery ward admissions increased from 4.5% in phase I to 28% in phase II, while internal medicine admissions decreased from 51.5% to 24.5% (Table 1).

**TABLE 1:** Demographic data.

Variable	Phase I (n = 200)	Phase II (n = 200)
Age (mean, years) [SD]	62.38 [17.0]	54.26 [20.1]
Length of stay (mean, days)	4.3	3.3
Females	89 (45%)	101 (50.5%)
Males	111 (55%)	99 (49.5%)
Ward—surgery	9 (4.5%)	56 (28%)
Ward—internal medicine	103 (51.5%)	49 (24.5%)
Ward—maternity	2 (1%)	30 (15%)
Ward—ICU	21 (10.5%)	36 (18%)
Ward—CCU	65 (32.5%)	29 (14.5%)

SD, standard deviation; ICU, intensive care unit; CCU, cardiac care unit.

A total of 84% of patients were prescribed PPIs on admission during phase I versus 78.5% during phase II. There were no significant changes in the route or type of PPI administration between both phases. Esomeprazole was the type of PPI predominantly prescribed in its IV form at 40 mg dose for both phases.

The indications for PPI use at admission, during hospitalization, and at discharge included the following: gastrointestinal reflux disease (GERD) with or without complications, peptic ulcer disease (PUD), functional dyspepsia, use of non-steroidal anti-inflammatory medications (NSAID) with any risk factors or with either of the previously mentioned indications, history of gastrointestinal bleeding, coronary artery disease (CAD) on aspirin or dual antiplatelets, and stress ulcer prophylaxis. The most common indication for both phases at admission and during hospital stay was CAD patients taking aspirin or dual antiplatelets (59 at admission), followed by patients on NSAIDs with risk factors for bleeding (23 at admission) (Table 2).

**TABLE 2:** PPI indications at admission by phase.

PPI indication at admission	Phase I (n = 109)		Phase II (n = 100)	
	Number of patients	Percentage	Number of patients	Percentage
CAD on ASA/DUAP	59	54%	48	48%
NSAID + risk factors	26	24%	38	38%
Stress ulcer prophylaxis	17	16%	19	19%
GI bleed	10	9%	0	-
GERD without complications	2	2%	4	4%
Functional dyspepsia	3	3%	2	2%
PUD	2	2%	0	-

Some patients had more than one documented PPI indication; therefore, categories are not mutually exclusive, and percentages may sum to more than 100%. CAD, coronary artery disease; ASA, aspirin; DUAP, dual anti-platelets; NSAID, non-steroidal anti-inflammatory drug; GI , gastro-intestinal; GERD, gastrointestinal reflux disease; PUD, peptic ulcer disease.

### PPI use among patients without a valid indication

In phase I, 44.5% of hospitalized patients had no valid indication for PPI use; among them, 77.5% were inappropriately prescribed a PPI, reflecting overuse (Table 3). In phase II, 63.5% of hospitalized patients had no valid indication for PPI prescription; of these, 69.3% were inappropriately prescribed a PPI, which also indicated overuse. These results show an 8.2% decrease in PPI prescription with no valid indication after the educational intervention, but this decrease was not statistically significant (p = 0.18).

**TABLE 3:** PPI prescription at admission by indication status.

​	Phase I (n = 200)	Phase II (n = 200)	p-value
Patients without a valid PPI indication			
Total without valid indication	n = 89	n = 127	​
PPI prescribed (inappropriate)	69 (77.5%)	88 (69.3%)	0.18
PPI not prescribed (appropriate)	20 (22.5%)	39 (30.7%)	0.18
Patients with a valid PPI indication			
Total with valid indication	n = 111	n = 73	​
PPI not prescribed (inappropriate)	12 (10.8%)	4 (5.5%)	0.21
PPI prescribed (appropriate)	99 (89.2%)	69 (94.5%)	0.21

Highlighted cells represent the primary outcome.

Among patients without a valid PPI indication (n = 216), multivariable logistic regression was performed to assess whether the difference in inappropriate PPI prescription was significant between the two phases after adjusting for age, sex, and ward category. After adjustment for age, sex, and ward category, the difference in inappropriate PPI prescription was not significant between the two phases (adjusted OR = 0.911, 95% CI: 0.454–1.82, p = 0.792), indicating that the observed crude decrease was not independently associated with the intervention (Table 4). Surgical ward admission was associated with significantly lower odds of inappropriate PPI prescription compared with non-surgical admission (adjusted OR = 0.402, 95% CI: 0.199–0.804, p = 0.010); otherwise, age and sex were not significantly associated with inappropriate PPI prescription in this restricted model.

**TABLE 4:** Multivariable logistic regression for inappropriate PPI prescription at admission among patients without a valid PPI indication (n = 216).

Variable	Adjusted OR	95% CI	p-value
Study phase			
Phase I (reference)	1.00	—	—
Phase II	0.911	0.454–1.82	0.792
Age (per year increase)	1.001	0.984–1.02	0.919
Sex			
Male (reference)	1.00	—	—
Female	0.811	0.437–1.51	0.505
Hospital ward			
Non surgery (reference)	1.00	—	—
Surgery	0.402	0.199–0.804	0.010*

OR, odds ratio; CI, confidence interval; ICU, intensive care unit; CCU, cardiac care unit. *p < 0.05; **p < 0.01.

### PPI use among patients with a valid indication

In phase I, 55.5% of the hospitalized patients had a valid indication for PPI use; among them, 10.8% were not prescribed a PPI, reflecting underuse (Table 3). In phase II, 36.5% of hospitalized patients had a valid indication for PPI prescription; of these, 5.5% were not prescribed a PPI, which also indicated underuse. These results show a 50.9% decrease in PPI underuse in patients with valid indication for PPI use after the educational intervention, but this decrease was also not statistically significant (p = 0.21).

## Discussion

The PPI prescription rates are high among hospitalized patients, with some studies reporting rates as high as 85% of patients (Sheikh-Taha et al., 2012). In our study, 84% of all hospitalized patients in phase I did receive a PPI at admission. In addition, 77.5% of patients without a valid indication for the use of PPI were inappropriately prescribed a PPI at admission, reflecting substantial overuse. Other studies demonstrated similarly high rates of PPI misuse. In one report, Souaid et al. showed that less than half of hospitalized patients (46.8%) received a PPI for an appropriate indication, with misuse rates particularly higher in surgical wards than in medical departments (Souaid et al., 2020). Another study demonstrated significant overuse of intravenous PPIs in non-ICU patients, with only 17% of those receiving stress ulcer prophylaxis meeting the guideline criteria, and a high proportion of inappropriate initiation occurs more frequently on medical floors than on surgical floors (Nasser et al., 2010). Misuse of PPI is also seen in the outpatient setting. An epidemiological study in Lebanon reported that 71.4% of outpatient PPI use was inappropriate, primarily due to incorrect indications, dosages, or treatment durations (Hoteit et al., 2020). Another Lebanese community-based study found that only 41.25% of outpatient PPI prescriptions for gastrointestinal bleeding prophylaxis complied with guideline-approved indications (Soubra and Issa, 2019). Underscoring the broader implications of inappropriate use, a large retrospective cohort study from Germany showed that unnecessary continuation of PPI therapy after ICU discharge was associated with higher risks of pneumonia, cardiovascular events, rehospitalization, and increased 2-year mortality (Del Giorno et al., 2018).

Post-intervention, the percentage of PPI overuse for admitted patients decreased from 77.5% to 69.3%, but the decrease was not statistically significant. The patient populations differed between the two phases, particularly with a much higher proportion of surgical ward patients in phase II. After adjusting for age, sex, and ward category, the difference in inappropriate PPI prescription was not significant between the two phases. Interestingly, patients in surgical wards were less likely to receive inappropriate PPI prescriptions compared with non-surgical patients, which is consistent with findings of Nasser et al.,(2010). Therefore, part of the observed reduction in inappropriate use of PPI during phase II may be related to the change in patient distribution between phases rather than to the educational intervention itself.

In contrast to the high rate of overuse for admitted patients described above, the rate of underuse, which is defined as the failure to prescribe a PPI when indicated, was only 10.8% in phase I. Interestingly, this rate decreased markedly in phase II after the intervention, but without statistical significance. Although underuse was less frequent than overuse, its clinical implications may still be significant, particularly given the potential risk of complications such as gastrointestinal bleeding when the indicated PPI therapy is omitted.

Developing and implementing de-prescription protocols in both hospital and community healthcare settings require collaboration among various professionals within a multidisciplinary management framework. Our intervention included multiple parties: hospitalist physicians, resident physicians in training, nurses, and pharmacists. The failure to achieve a significant decrease in PPI overuse reflects resistance to change among the different players involved in PPI prescription. Our intervention was limited to an educational tool, with the final PPI prescribing decisions left to the treating team and no further follow-up of individual decision-making. This was intentionally designed to assess the impact of a single non-coercive educational intervention on prescribing behavior, which is in contrast to antibiotic stewardship programs, where prescriptions often require approval by a specialized team.

Our findings reflect the magnitude of the problem and the need for stricter interventions to achieve a change in behavior. Hospital pharmacists play a crucial role in therapeutic reconciliation upon admission by identifying cases where the use of PPI is not indicated and should be involved in de-prescription. Effective communication between healthcare professionals across different levels of care, along with proper documentation in medical records, is essential to enable other professionals to make de-prescription decisions. PPI stewardship should be discussed and implemented as seriously and strictly as antibiotic stewardship due to its medical and financial burdens.

Our study has several limitations. The significant heterogeneity in ward distribution between the two phases, most notably, the increase in surgical admissions, represents a potential confounding source. Because some individual ward categories had small or empty subgroups, a simplified surgery versus non-surgery ward classification was used, which may not fully capture ward-specific prescribing patterns. Additionally, because no formal pre- and post-intervention knowledge assessment was conducted among participating healthcare staff, we could not determine whether the educational session effectively improved knowledge of appropriate PPI prescribing indications or the extent to which the remaining knowledge gaps contributed to the persistent inappropriate prescribing observed in phase II. Future interventional studies should incorporate validated knowledge assessments to better characterize the relationship between educational impact and prescribing behavior change. Finally, the single-center design at a community hospital in Lebanon may limit the generalizability of these findings to other healthcare settings.

## Conclusion

This study demonstrated that a non-coercive targeted quality of care improvement intervention is not sufficient to significantly reduce the overuse of PPI among hospitalized patients. More strict and continued efforts in education and adherence to guidelines are essential to achieve significant results. Future studies should focus on long-term outcomes of such interventions and their impact on patient health and healthcare costs.

## Data Availability

The original contributions presented in the study are included in the article/supplementary material; further inquiries can be directed to the corresponding author.
