# Correction: Proton pump inhibitor prescription patterns in hospitalized patients before and after targeted intervention

**DOI:** 10.3389/fphar.2026.1944589

**Published:** 2026-09-24

**Authors:** Johny Gerjy Salem, Sarah Jalloul, Ahmad Hammoud, Jihane Atrash, Karim Halabi, Melissa Alkatrib, Mira Sabat, Louis Chaptini

**Affiliations:** 1 Department of Gastroenterology, Faculty of Medicine, University of Balamand, El Koura, Lebanon; 2 Department of Internal Medicine, Faculty of Medicine, University of Balamand, El Koura, Lebanon; 3 Department of Mathematics, Faculty of Arts and Sciences, University of Balamand, El Koura, Lebanon; 4 Department of Medicine, Digestive Diseases, Yale School of Medicine, New Haven, CT, United States

**Keywords:** inappropriate prescription, Lebanon, overuse, proton pump inhibitor, stewardship

The incorrect city was provided for University of Balamand. The correct city is El Koura.

Department details were omitted from the affiliations. Affiliation 1 has been updated as “Department of Gastroenterology, Faculty of Medicine, University of Balamand, El Koura, Lebanon”.

Affiliation 2 “Department of Internal Medicine, Faculty of Medicine, University of Balamand, El Koura, Lebanon” was omitted from the paper due to the omission of the department details. This affiliation has now been added for authors Ahmad Hammoud, Jihane Atrash, Karim Halabi, Melissa Alkatrib. Note that these authors are no longer associated with Affiliation 1, as it is the wrong department.

Affiliation 3 “Department of Mathematics, Faculty of Arts and Sciences, University of Balamand, El Koura, Lebanon” was omitted from the paper due to the omission of the department details. This affiliation has now been added for author Mira Sabat. Note that this authors is no longer associated with Affiliation 1, as it is the wrong department.

Department details were omitted from the affiliations. Affiliation 4 (previously published as Affiliation 2) has been updated as “Department of Medicine, Digestive Diseases, Yale School of Medicine, New Haven, CT, United States”.

The original version of this article has been updated.

